# Effects of reduced β_2_-glycoprotein I on the expression of aortic matrix metalloproteinases and tissue inhibitor matrix metalloproteinases in diabetic mice

**DOI:** 10.1186/1471-2261-14-114

**Published:** 2014-09-10

**Authors:** Jun Xu, Penghua Wang, Tong Wang, Meijun Wang, Sisi Chen, Pei Yu, Demin Yu

**Affiliations:** 2011 Collaborative Innovation Center of Tianjin for Medical Epigenetics, the Key Laboratory of Hormones and Development (Ministry of Health), Metabolic Diseases Hospital & Tianjin Institute of Endocrinology, Tianjin Medical University, No.66 Tong-An Road, Heping district, Tianjin, 300070 China

**Keywords:** Reduced β_2_GP I, Diabetes, Aorta, MMP, TIMP, p38MAPK, Signaling pathway

## Abstract

**Background:**

Reduced β_2_-glycoprotein I (reduced β_2_GP I), which has free sulfhydryl groups, is present in plasma and serum; it can protect vascular endothelial cells from damage due to oxidative stress *in vitro.* We investigated the effects of reduced β_2_GP I on the expression of various matrix metalloproteinases (MMPs) and tissue inhibitors of matrix metalloproteinases (TIMPs) in the aortas of diabetic mice.

**Methods:**

We provided 120 female 8-week-old Balb/c mice with a high sugar, high fat diet. After 8 weeks they were injected with streptozotocin to induce diabetes. We treated mice in the mono dose groups with β_2_GP I, reduced β_2_GP I, or phosphate-buffered saline (PBS) on day 1 and fed them for 3 weeks. The mice in the complex dose groups were treated with β_2_GP I, reduced β_2_GP I, or PBS on days 1 and 22 and fed for 6 weeks. Control mice were given a standard chow diet. Blood lipids were measured at the end of 3 or 6 weeks, and aortas removed to observe morphological and molecular biological changes.

**Results:**

The low-density lipoprotein cholesterol levels in mice of the reduced β_2_GP I group were lower than those in the diabetic group. Aortic lipid deposition in the reduced β_2_GP I group was significantly less than in the diabetic control group. In the aortas, reduced β_2_GP I decreased MMP2/TIMP2 mRNA and protein expression levels, and MMP9/TIMP1 expression levels compared with those in diabetic controls. Reduced β_2_GP I down-regulated p38 mitogen-activated protein kinase (p38MAPK) mRNA expression and phosphorylated p38MAPK protein expression compared with those in diabetic controls of the complex dose group.

**Conclusions:**

Reduced β_2_GP I plays a role in diabetic mice related to vascular protection, inhibiting vascular lipid deposition, and plaque formation by reducing MMPs/TIMPs expression through down-regulation of the p38MAPK signaling pathway.

## Background

The latest epidemiological data show that the prevalence of diabetes is 11.6%, and pre-diabetes is 50.1%, in people over 18 years old in China [1]. The macrovascular complications of diabetes mainly cause damage to the cardiovascular system. Diabetes is related to a higher risk of coronary heart disease, especially in patients who have had diabetes for more than 10 years [2]. Patients with diabetes are also more prone to atherosclerosis and cardiovascular events (angina, myocardial infarction, heart failure) [3, 4]. However, the pathogenesis of diabetes has not been fully elucidated. β_2_-glycoprotein I (β_2_GP I) is a phospholipid-binding plasma protein, and an autoantigen. In patients with type 2 diabetes or myocardial infarction, plasma β_2_GP I and oxidized low-density lipoprotein (oxLDL)/β_2_GP I complex levels are significantly increased; these are predicted adverse consequences of cardiovascular events [5]. Previous studies have shown that β_2_GP I/oxLDL/C reactive protein (CRP) complexes can up-regulate the expression of p38MAPK, increasing the generation of atherosclerosis in diabetic mice [6]. Reduced β_2_GP I, which has free sulfhydryl groups, is also present in plasma and serum; it can protect endothelial cells from damage due to oxidative stress [7]. Matrix metalloproteinases (MMPs) can degrade all extracellular matrix components, resulting in their increased activity in the aortic plaque. They can then degrade collagen fibers, making the fibrous cap thin and plaques easily broken. Type IV collagen is an important component of the basement membrane in atherosclerotic plaques and fibrous caps. Gelatinases (MMP2, MMP9) responsible for its degradation encourage smooth muscle cells to migrate into the intima membrane and accelerate atherosclerosis, leading to unstable plaque formation [8]. Oxidative stress is present in diabetes, and elevated levels of reactive oxygen species can lead to elevated MMP2 and MMP9 levels [9]. Tissue inhibitors of matrix metalloproteinases (TIMPs) are natural inhibitors of MMPs; TIMP-1 can inhibit MMP9, while TIMP-2 can inhibit MMP2. The TIMPs are known suppressors of atherosclerosis [10, 11]. Previous studies have shown that reduced β_2_GPI can inhibit the formation of foam cells by macrophages and apoptosis *in vitro* [12]. The aim of our study was to investigate how reduced β_2_GP I by MMPs/TIMPs affect the aorta *in vivo*, and to determine any related mechanisms of action involved.

## Methods

### Animal models and groups

All animal experiments were approved by the Animal Care and Research Committee of Tianjin Medical University. All procedures were performed in accordance with the Guidelines of Animal Experiments from the Committee of Medical Ethics, the National Health Department of China (1998). We obtained 160 female Balb/c mice (8 weeks old) weighing 18–25 g were obtained from the Peking University Experimental Animal Center. We randomly selected 40 mice as the normal control group; these mice were given a standard chow diet for 8 weeks and injected with sodium citrate buffer. The remaining 120 mice were given a high sugar and high fat diet (10% sugar, 10% lard, 5% yolk, 1% cholesterol, and 0.2% bile salt by mg) for 8 weeks. These mice were then intraperitoneally injected with 80 mg/kg of 2% streptozotocin twice. Tail vein blood glucose levels were measured one week later; mice with a blood glucose concentration ≥ 16.7 mM were considered diabetes.

Diabetic mice were randomly divided into six groups (*n* = 20 mice per group). There were three mono-dose groups that were injected once in the tail vein on day 1: the β_2_GP I group (20 μg); the reduced β_2_GP I group (20 μg); and the diabetic control group treated with phosphate-buffered saline (PBS). We used PBS as the vehicle for β_2_GP I and reduced β_2_GP I. We also had three complex-dose groups that were injected twice in the tail vein on days 1 and 22: the β_2_GP I group (20 μg each injection), the reduced β_2_GP I group (20 μg each injection); and the diabetic control group (PBS). The 40 normal control mice were randomly divided into two groups (*n* = 20 mice per group), so that there were controls for the mono- and complex-dose groups, and injected with PBS.

### Body weight and blood glucose

Body weight was assessed every week. Following injection with streptozotocin, blood glucose levels were monitored weekly.

### Specimen collection

Blood from mice in the mono-dose groups were sampled at day 22, and at day 43 for those in the complex-dose groups. Blood was obtained via retro-orbital plexus and mice were sacrificed by cervical dislocation. Aortas were carefully dissected from the iliac bifurcation to the aortic arch and external fatty deposits were removed. Complete aortas were then collected.

### Determination of serum lipids

Blood samples were centrifuged (3500 rpm, 5 min, room temperature) and the plasma concentration of triglycerides, total cholesterol, low density lipoprotein cholesterol (LDL-c), and high-density lipoprotein cholesterol (HDL-c) were determined by enzymatic colorimetric assays using an Automatic Biochemical Analyzer (Hitachi Co., Japan).

### Aortic lipid analysis

Aortas were fixed in 4% (w/v) paraformaldehyde overnight and cut open longitudinally. After rinsing in 70% (v/v) ethanol, specimens were stained with Sudan IV solution for 15 min and decolorized in 80% (v/v) ethanol for 20 min until the normal tissue turned white. Neutral resins were used to block specimens. Images were acquired using a PowerShot S70 camera (Canon, Japan) and analyzed with ImageJ 2.1.4.7 (National Institutes of Health, USA). We then calculated the percentage of plaque coverage.

### Histopathology analysis

Paraffin-embedded aortas were serially sectioned (5 μm thickness) and deparaffinized with dimethylbenzene (2 × 15 min), then treated with absolute ethanol (2 × 5 min), washed with distilled water, and stained with hematoxylin and 0.5% eosin. Aortas were washed with distilled water, then placed through a graded series of ethanol [80, 95 and 100% (v/v)], incubated with dimethylbenzene (2 × 2 min), and blocked with neutral resins. Sections were observed using microscopy to compare histopathological alterations in the various groups.

### Quantitative polymerase chain reaction assays

The oligonucleotide primer sequences for amplification and quantitation of TIMP-1, TIMP-2, MMP2, MMP9, p38MAPK, and glyceraldehyde-3-phosphate dehydrogenase (GADPH) are presented in Table 1. Total RNA was isolated using Trizol (Sigma-Aldrich, USA). We used a reverse transcription kit to synthesize RNA into cDNA. For quantitative polymerase chain reaction assays, each reaction comprised 5 μL of SYBR® Green II, 0.4 μL of each downstream and upstream, 1 μL of cDNA, and 3.2 μL of diethylpyrocarbonate-treated water. Thermal cycling conditions involved incubation at 50°C for 2 min followed by 94°C for 3 min, then 45 cycles of 94°C for 30 s, 30 s at the appropriate annealing temperature (64.5°C for MMP2 and MMP9; 61.4°C for TIMP-1, TIMP-2, and p38MAPK; and 58°C for GADPH), and 75°C for 45 s. After the 45th cycle, samples were incubated at 72°C for 10 s, then at 65°C for 5 s, and the temperature raised to 95°C to complete the assay. The fold change in mRNA expression levels were assessed using the 2^-ΔΔCt^ method [ΔΔCt = (Ct1 - Ct2) - (Ct3 - Ct4)]. Ct1 and Ct2 represent the critical cycle numbers for the target gene and GADPH, respectively, in the β_2_GP I, reduced β_2_GP I and diabetic control groups, respectively. Ct3 and Ct4 represent the critical cycle numbers for the target gene and GADPH, respectively, in the normal control group.

**Table 1:** **Primer sequence of TIMP-1, -2, MMP2, MMP9, p38MAPK and GAPDH for real time PCR**

Gene name	Primer sequence	
TIMP-1	Forward	5′AGACACACCAGAGCAGATACC3′
	Reverse	5′CAGCTACAGGCCTTACTGGAA3′
TIMP-2	Forward	5′GCTCCAACCCTGTCCTAACC3′
	Reverse	5′GCACAACACGAAAATGCCCT3′
MMP2	Forward	5′TTTCTATGGCTGCCCCAAGG3′
	Reverse	5′GTCAAGGTCACCTGTCTGGG3′
MMP9	Forward	5′CGGATCCCCAACCTTTTCCA3′
	Reverse	5′GTGCCTGTCACAAAAGCCAG3′
p38 MAPK	Forward	5′AAGACTCGTTGGAACCCCAG3′
	Reverse	5′GGGTCGTGGTACTGAGCAAA3′
GADPH	Forward	5′CAAGGTCATCCATGACAACTTG3′
	Reverse	5′GTCCACCACCCTGTTGCTGTAG3′

### Western blotting assays

Aortas were dissolved and the concentration of total protein was determined using BCA reagents (thermo scientific, USA) according to the manufacturer’s instructions. We added 30 μg of sample protein per 20 μL to a sodium dodecyl sulfate polyacrylamide gel electrophoresis buffer. The concentration of polyacrylamide gels used was dependent on the molecular weight of the protein examined. Gel concentrations were 10% for MMP2 (72 kDa, Proteintech, USA), p38MAPK and phosphorylated p38MAPK (38 kDa, Cell Signaling Technology, USA), β-tubulin (55 kDa, Sigma, USA), and 15% for TIMP-1 (28 kDa, Proteintech, USA), TIMP-2 (21 kDa, Santa Cruz, USA). Proteins were electrophoresed (110 V) and transferred to nitrocellulose membranes, then incubated at room temperature with 5% (w/v) skim milk in Tris-buffered saline with Tween 20 (TBST) for 1.5 h. Membranes were incubated overnight at 4°C with antibodies against MMP2, TIMP-1, p38MAPK, phosphorylated p38MAPK (all diluted 1:1000), TIMP-2 (1:100), and β-tubulin (1:5000) diluted in TBST. Membranes were washed with TBST (3 × 15 min) and incubated at room temperature with the appropriate secondary antibody (1:20,000) for 1 h. Membranes were washed with TBST (5 × 10 min) and immunoreactive bands were detected using enhanced chemiluminescence reagents followed by image analysis with ImageJ.

### Statistical analysis

We used SPSS19.0 to analyze our data, with values expressed as mean ± standard deviation. Analysis of variance was used for mono- and complex-dose groups. If there was statistical significance then we conducted post-hoc analyses with the diabetic control group as a reference using Dunnett’s test. A *P*-value less than 0.05 was considered statistically significant.

## Results

### Blood glucose and body weight

The weight of mice in the diabetic groups declined and then gradually increased (data not shown). Table 2 shows the blood glucose levels and body weight of mice the day before they were sacrificed. There was no difference in body weight among groups (*P* = 0.47 in mono-dose, *P* = 0.43 in complex-dose). Blood glucose levels in mice of the diabetic groups were significantly higher than those in the normal control group (*P* = 0.03 in mono-dose, *P* = 0.02 in complex-dose), with no difference for mice in the diabetic groups (*P* = 0.51 in mono-dose, *P* = 0.35 in complex-dose).

**Table 2:** **Changes in blood glucose and body weight**

Groups	n	Blood glucose (mM)		Body weight (g)	
		Mono dose	Complex dose	Mono dose	Complex dose
β_2_GP I	40	24.11 ± 7.54*	20.86 ± 6.96*	26.13 ± 2.36	25.85 ± 3.32
Reduced β_2_ GP I	40	24.07 ± 11.20*	18.19 ± 6.43*	25.50 ± 3.42	27.10 ± 2.90
Diabetic control	40	26.07 ± 4.42*	19.00 ± 7.75*	23.67 ± 3.20	24.60 ± 3.34
Normal control	40	7.40 ± 1.12	5.93 ± 0.79	24.67 ± 2.08	27.73 ± 2.97

^*^ *P* < 0.05 vs. normal control.

### Reduced β_2_GP I diminished LDL-c levels

**Figure 1:**
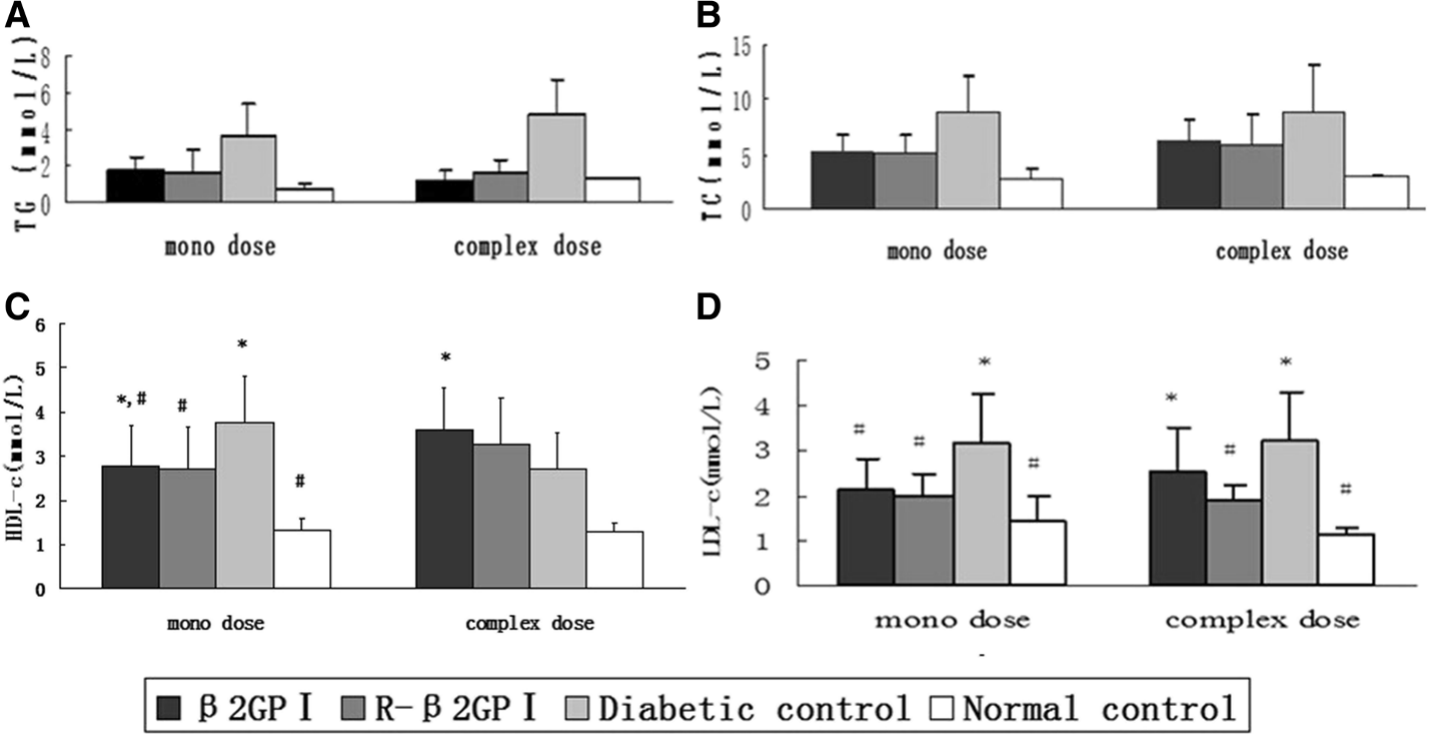
**Blood lipid changes in each group.** We obtained 160 female Balb/c mice (8 weeks old) and randomly selected 40 mice as the normal control group, which were given a standard chow diet for 8 weeks. The remaining 120 mice were given a high sugar and high fat diet for 8 weeks. These mice were then intraperitoneally injected with 80 mg/kg of 2% streptozotocin twice. Mice with a blood glucose concentration ≥ 16.7 mM were considered diabetes. Diabetic mice were randomly divided into six groups (*n* = 20 mice per group). There were three mono-dose groups that were injected once in the tail vein on day 1: the β_2_GP I group (20 μg); the reduced β_2_GP I group (20 μg); and the diabetic control group treated with PBS. We used PBS as the vehicle for β_2_GP I and reduced β_2_GP I. We also had three complex-dose groups that were injected twice in the tail vein on days 1 and 22: the β_2_GP I group (20 μg each injection), the reduced β_2_GP I group (20 μg each injection); and the diabetic control group (PBS). The 40 normal control mice were randomly divided into two groups (*n* = 20 mice per group), so that there were controls for the mono- and complex-dose groups, and injected with PBS. The blood lipids were tested at day 22 in mono-dose groups and at day 43 in complex-dose groups. **A**. Plasma concentration of triglycerides (TG). **B**. Plasma concentration of total cholesterol (TC). **C**. Plasma concentration of low-density lipoprotein cholesterol (LDL-c). **D**. Plasma concentration of high-density lipoprotein cholesterol (HDL-c). Values are presented as mean ± SD. ^*^ *P* < 0.05 vs. normal controls; ^#^ *P* < 0.05 vs. diabetic controls; ^@^ *P* < 0.05 vs. reduced β_2_GP I (R-β_2_GP I); and &*P* < 0.05 vs. β_2_GP I.

Lipid indicators were highest in the diabetic control group, with total cholesterol (*P* = 0.08 in mono-dose, *P* = 0.12 in complex-dose) and triglycerides (*P* = 0.22 in mono-dose, *P* = 0.09 in complex-dose) not statistically significant (Figure 1A–B). Although HDL-c levels in the β_2_GP I and reduced β_2_GP I mice of the complex-dose groups were higher than those in the diabetic control group, these were not statistically significant (*P* = 0.23 in mono-dose, *P* = 0.15 in complex-dose; Figure 1C). LDL-c levels in the reduced β_2_GP I mice of mono- and complex-dose groups were lower than those in the diabetic control group (*P* = 0.02 in mono-dose, *P* = 0.01 in complex-dose; Figure 1D). LDL-c levels were lower in the β_2_GP I mono-dose group compared with those in the diabetic control group (*P* = 0.04; Figure 1D).

### Aortic lipid analysis

**Figure 2:**
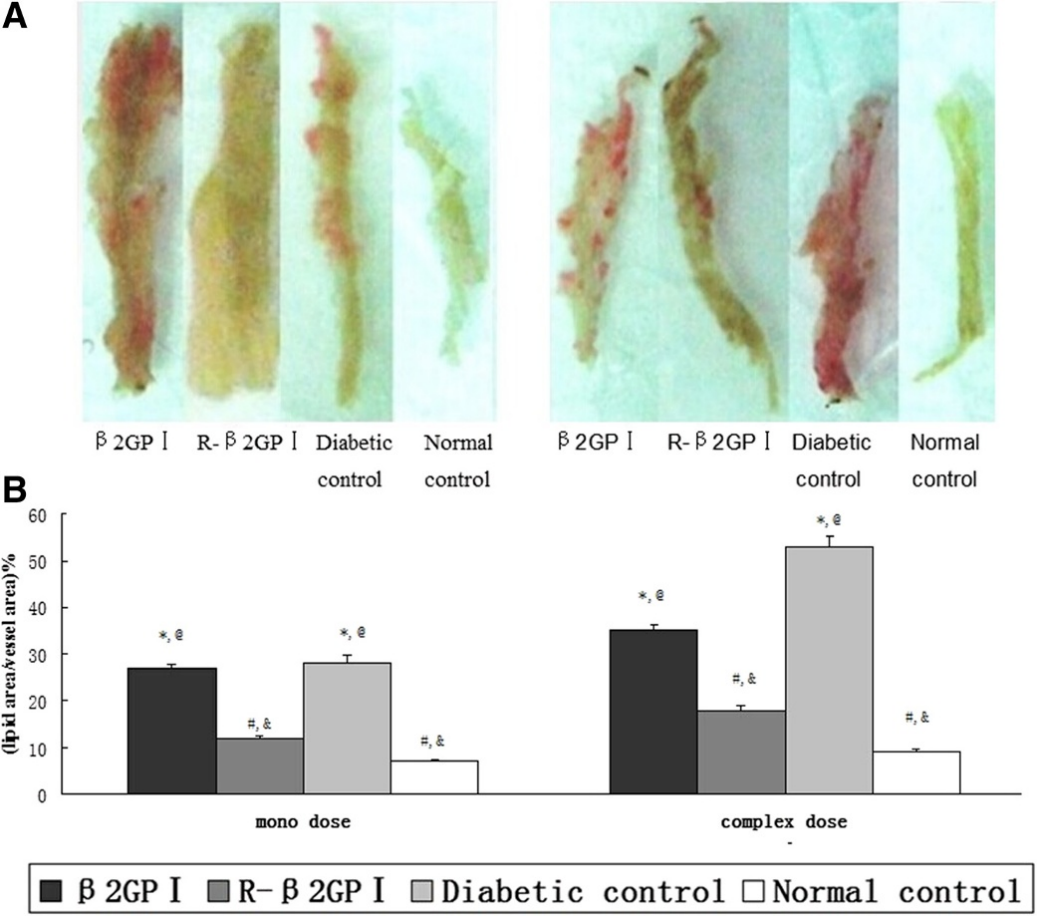
**Aortic lipid staining with Sudan IV. A**. Aortas were fixed in 4% (w/v) paraformaldehyde overnight and cut open longitudinally. After rinsing in 70% (v/v) ethanol, specimens were stained with Sudan IV solution for 15 min and decolorized in 80% (v/v) ethanol for 20 min until the normal tissue turned white. Neutral resins were used to block specimens. Images were acquired using camera and analyzed with ImageJ. We then calculated the percentage of plaque coverage. Red staining is indicative of lipid deposition. **B**. Lipid area/total vessel area for five aortas selected from each group. ^*^ *P* < 0.05 vs. normal controls; ^#^ *P* < 0.05 vs. diabetic controls; ^@^ *P* < 0.05 vs. reduced β_2_GP I (R-β_2_GP I); and &*P* < 0.05 vs. β_2_GP I.

From the aortic cross-sectional view, there was obvious red in the diabetic control group, indicative of lipid deposition. Lipid deposition was also seen in the arterial walls of mice in the β_2_GP I group (complex-dose). There was no significant lipid deposition in mice of the reduced β_2_GP I and normal control groups (Figure 2A). Aortic lipid deposition in the reduced β_2_GP I group was significantly less than that in the diabetic control group (*P* = 0.01 in mono-dose, *P* = 0.01 in complex dose; Figure 2B). Aortic lipid deposition in mice of the β_2_GP I group was more pronounced than in the reduced β_2_GP I group (*P* = 0.01 in mono-dose, *P* = 0.03 in complex-dose; Figure 2B).

### Morphological changes in aortas

**Figure 3:**
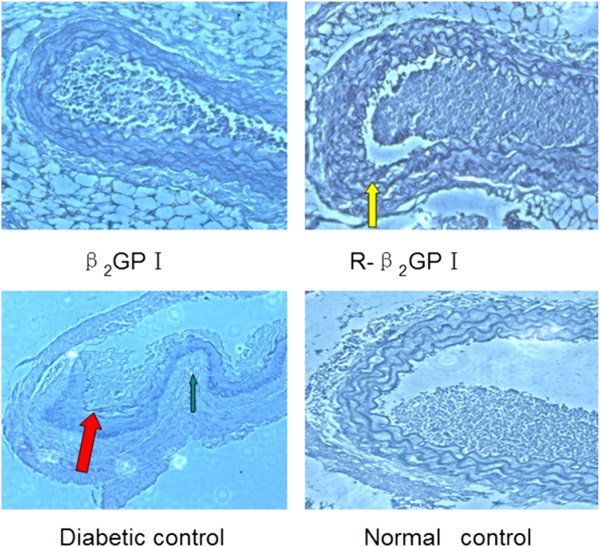
**Morphological changes in blood vessels for the complex-dose groups.** Aortas were carefully dissected from the iliac bifurcation to the aortic arch and external fatty deposits were removed. Complete aortas were collected at day 43 (complex-dose group), with five aortas selected from each group, and observed by light microscopy following HE staining (200× magnification). In the diabetic control group, the vascular wall had thickened and atherosclerotic plaques were present. Plaque surfaces had a fibrous cap (red arrow). Under the fibrous cap, there were a large number of foam cells along with formation of white cholesterol crystals (green arrow). In the reduced β_2_GP I group, the structure of the vessel wall was intact (yellow arrow), with no thickening of the endothelium and no significant foam cell formation.

There were no significant vascular morphological changes in the mono-dose groups (data not shown). In the diabetic control mice from the complex-dose group, aortic lipid plaques were seen as evidenced by fibrous cap formation. Many foam cells were seen under the fibrous cap. In the reduced β_2_GP I group the structure of the vessel wall was intact, the endothelium showed no thickening, and there was no significant foam cell formation (Figure 3).

### Effects of reduced β_2_GP I on aortic mRNA and protein expression levels

**Figure 4:**
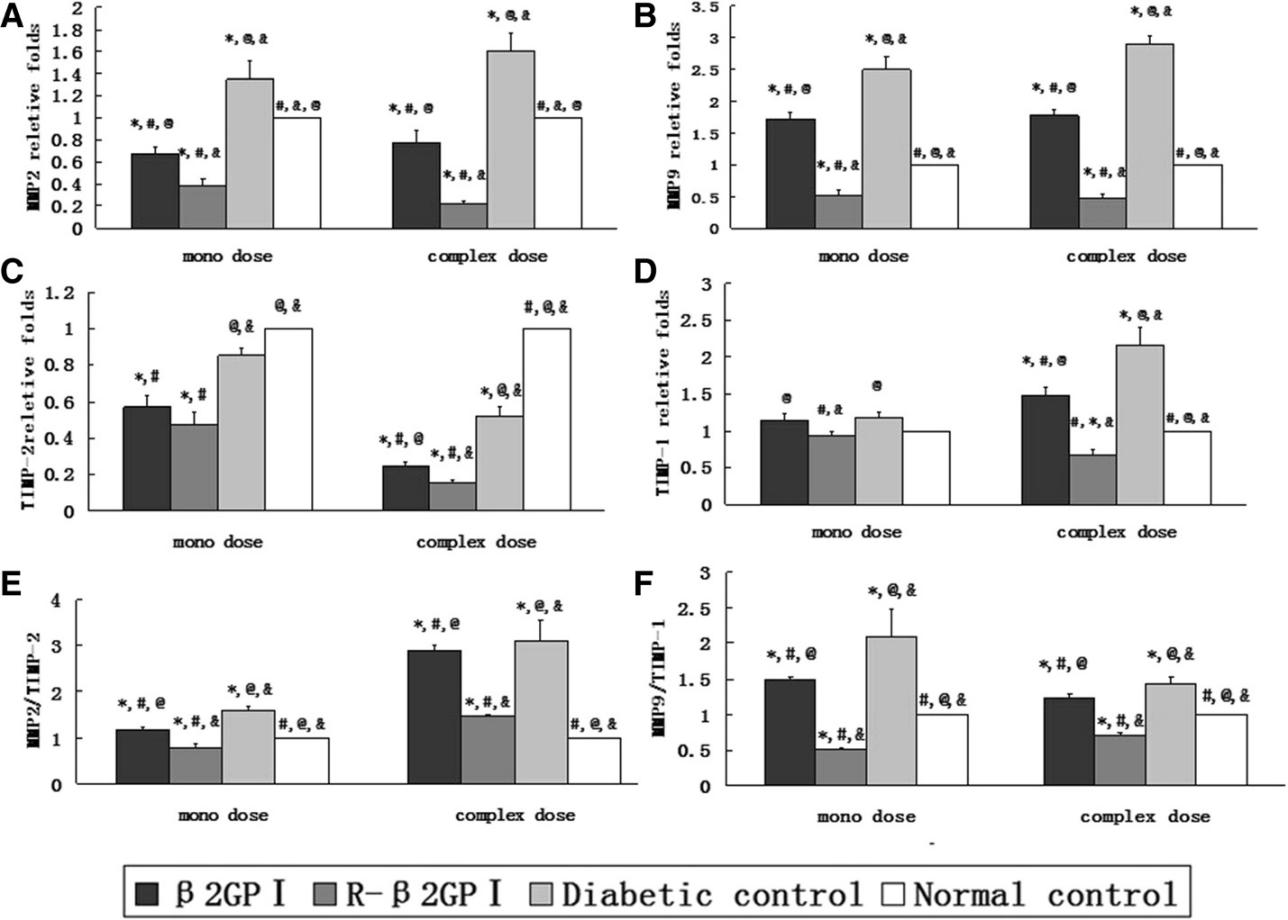
**Effects of reduced β**_**2**_ **GP I on aortic MMP2, MMP9, TIMP-1, and TIMP-2 mRNA expression levels.** Aorta was sampled at day 22 (mono-dose groups) and day 43 (complex-dose groups) with ten aortas selected from each group after treatment and then assessed by real time PCR. GADPH was selected as the internal control. **A**. MMP2. **B**. MMP9. **C**. TIMP-2. **D**. TIMP-1. **E**. MMP2/TIMP-2. **F**. MMP9/TIMP-1. ^*^ *P* < 0.05 vs. normal controls; ^#^ *P* < 0.05 vs. diabetic controls; ^@^ *P* < 0.05 vs. reduced β_2_GP I (R-β_2_GP I); and &*P* < 0.05 vs. β_2_GP I.

The mRNA expression levels for MMP2, MMP9, and TIMP-1 were highest in mice of the diabetic control groups (MMP2: *P* = 0.03 in mono-dose, *P* = 0.01 in complex-dose, Figure 4A; MMP9: *P* = 0.02 in mono-dose, *P* = 0.01 in complex-dose, Figure 4B; TIMP-1: *P* = 0.04 in mono-dose, *P* = 0.03 in complex-dose, Figure 4D). Reduced β_2_GP I down-regulated MMP2, MMP9, TIMP-1, TIMP-9 mRNA expression. For the complex-dose group at day 42, expression was lowest (MMP2: *P* = 0.01; MMP9: *P* = 0.01; TIMP-1, *P* = 0.02; TIMP-9: *P* = 0.03; Figure 4A–D). MMP2/TIMP2 and MMP9/TIMP1 expression ratios showed that reduced β_2_GP I could suppress the system; these were decreased by 50.3% and 75.2% respectively for the mono-dose group, and 52.6% and 50.3% respectively for the complex-dose group compared with diabetic controls. β_2_GP I also reduced MMP2/TIMP2 and MMP9/TIMP ratios by 25.8% and 28.6% (mono-dose) respectively, and 6.5% and 13.3% (complex-dose) respectively compared with diabetic controls (MMP2/TIMP2: *P* = 0.04 in mono-dose, *P* = 0.03 in complex-dose; MMP9/TIMP1: *P* = 0.03 in mono-dose; *P* = 0.04 in complex-dose; Figure 4E–F).

**Figure 5:**
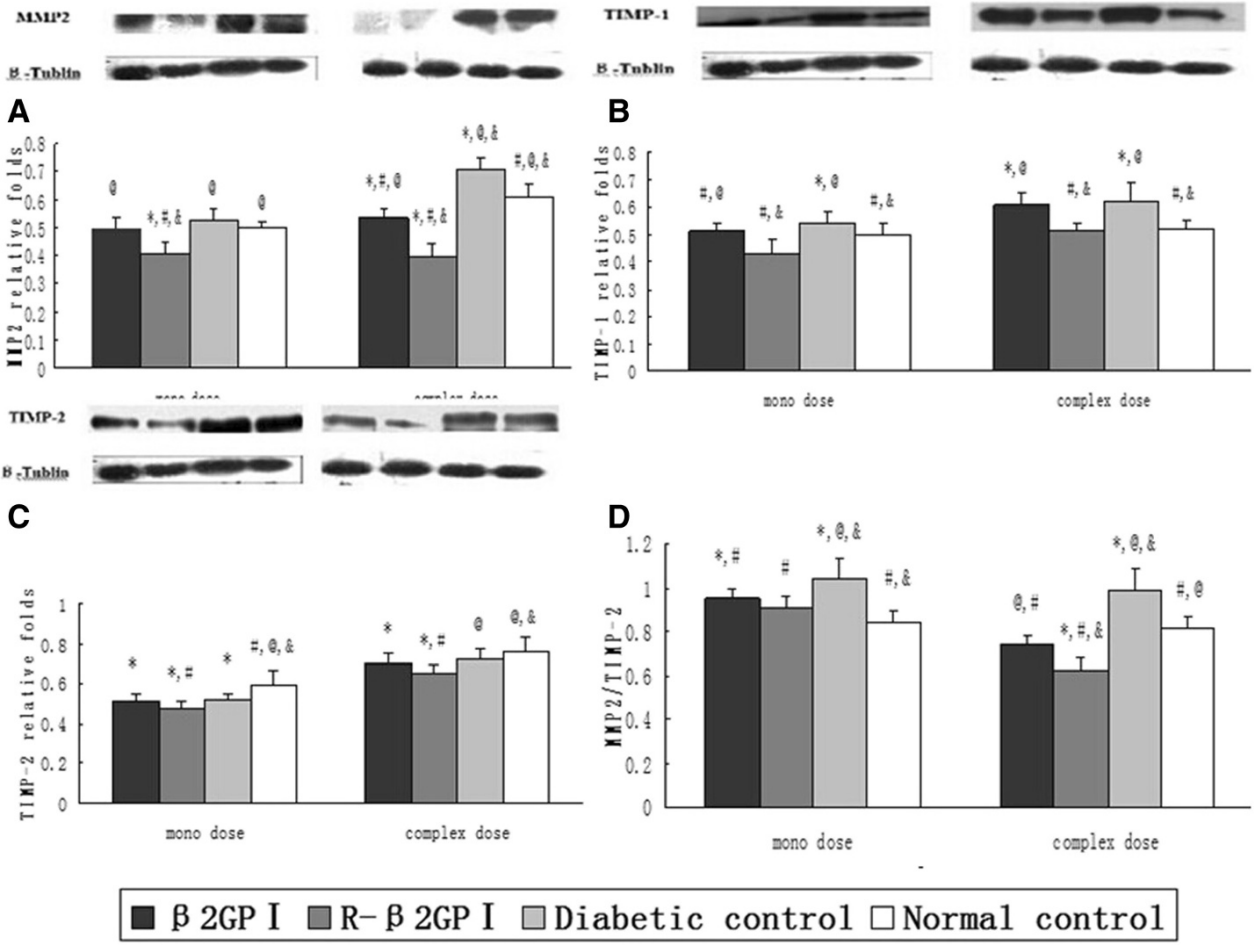
**Effects of reduced β**_**2**_ **GP I on aortic MMP2, TIMP-1, and TIMP-2 protein expression levels.** Aorta was sampled at day 22 (mono-dose groups) and day 43 (complex-dose groups) with ten aortas selected from each group after treatment and then assessed by Western Blotting. β-Tublin was selected as the internal control. **A**. MMP2. **B**. TIMP-1. **C**. TIMP-2. **D**. MMP2/TIMP-2. ^*^ *P* < 0.05 vs. normal controls; ^#^ *P* < 0.05 vs. diabetic controls; ^@^ *P* < 0.05 vs. reduced β_2_GP I (R-β_2_GP I); and &*P* < 0.05 vs. β_2_GP I.

MMP2, TIMP-1, TIMP-2 protein expression levels for mono-dose and complex-dose mice in the reduced β_2_GP I group were lower than those in the diabetic control group (MMP2: *P* = 0.04 in mono-dose, *P* = 0.03 in complex-dose, Figure 5A; TIMP-1: *P* = 0.04 in mono dose, *P* = 0.04 in complex dose, Figure 5B; TIMP-2: *P* = 0.04 in mono-dose, *P* = 0.04 in complex-dose, Figure 5C). MMP2/TIMP-2 ratios in the reduced β_2_GP I group were decreased by 12.5% (mono-dose) and 37.4% (complex-dose) compared with the diabetic control group, decreased by 26.2% (complex-dose) compared with the normal control group, and decreased by 16.2% (complex-dose) compared with the β_2_GP I group (MMP2/TIMP-2: *P* = 0.04 in mono-dose, *P* = 0.03 in complex-dose; Figure 5D).

### Reduced β_2_GP I down-regulated the p38MAPK signaling pathway in aortas

**Figure 6:**
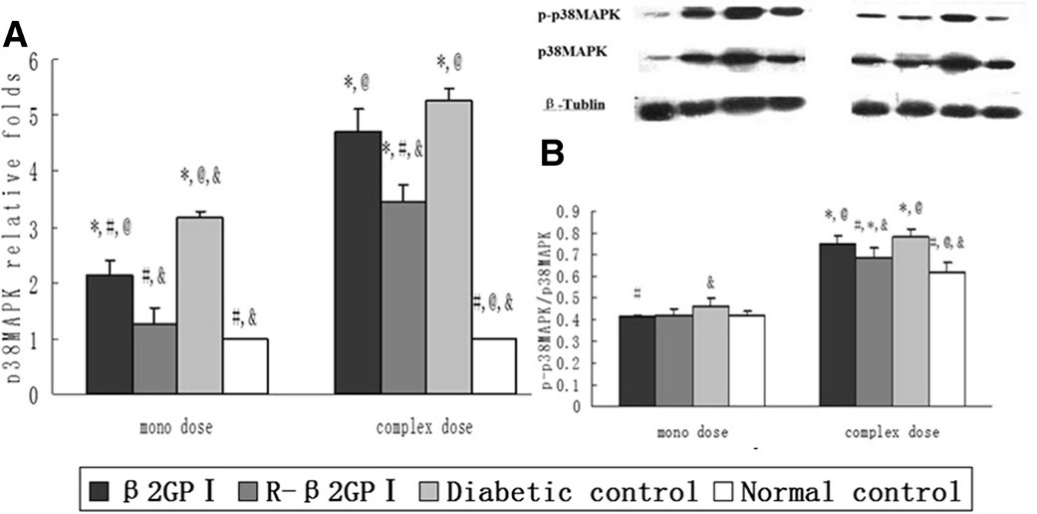
**Reduced β**_**2**_ **GP I down-regulated the p38MAPK signaling pathway in aortas.** Aorta was sampled at day 22 (mono-dose groups) and day 43 (complex-dose groups) with ten aortas selected from each group after treatment and then assessed by real time PCR (GADPH was selected as the internal control) and Western Blotting (β-Tublin was selected as the internal control). **A**. Relative fold change in p38MAPK mRNA levels. **B**. Ratio of phosphorylated p38MAPK to p38MAPK protein. ^*^ *P* < 0.05 vs. normal control; ^#^ *P* < 0.05 vs. diabetic controls; ^@^ *P* < 0.05 vs. reduced β_2_GP I (R-β_2_GP I); and &*P* < 0.05 vs. β_2_GP I.

Expression of p38MAPK mRNA and protein were increased in the diabetic control group. Expression of p38MAPK mRNAs in the reduced β_2_GP I group were down-regulated by 71.9% (mono-dose) and 80.3% (complex-dose) compared with those in the diabetic control group (*P* = 0.01 in mono-dose, *P* = 0.03 in complex-dose; Figure 6A). The ratio of phosphorylated p38MAPK protein to total p38MAPK protein in the reduced β_2_GP I group was decreased by 11.5% (complex-dose) compared with those in the diabetic control group (*P* = 0.04; Figure 6B).

## Discussion

β_2_GP I and oxLDL-c can form a complex in the body that acts as an antigen to promote arteriosclerosis. β_2_GP I and CD4^+^ lymphocytes and monocyte-derived macrophages co-localize to human atherosclerotic sites. This indicates that oxLDL-c/β_2_GP I and β_2_GP I antibodies are higher in acute coronary syndrome patients as adverse reactions increase [13]. Our previous study results have shown that oxLDL-c/β_2_GP I /CRP complexes promote macrophages that have internalized oxLDL-c to form foam cells. This accelerates the formation of atherosclerosis in diabetic mice [6]. Results from our previous study also showed that endothelial cells can secrete thioredoxin-1 to change β_2_GP I into reduced β_2_GP I. This is generated when the functional disulfide (Cys288-Cys326) is opened and free sulfhydryl groups are present in domain V, playing a role in endothelial protection during oxidative stress *in vitro*. We also found that β_2_GP I and reduced β_2_GP I are present in the plasma [7]. Studies have shown elevated β_2_GP I levels in the plasma of type 2 diabetic patients [14]. However, another study showed that the level of β_2_GP I was not different between diabetic and non-diabetic patients matched for age, sex and body mass index. These results indicated that β_2_GP I levels rose in diabetic patients with obesity and metabolic syndromes [15].

When plaques are present in blood vessels of diabetic patients, the greatest hazard is plaque rupture [16]. A reason for plaque instability is its thin fibrous cap. MMPs are a class of widespread endopeptidases, whose main role is to break down the extracellular matrix. Collagen IV is an important component at the bottom of the plaque base and the fibrous cap. Collagen IV is degraded by MMP2 and MMP9, resulting in vascular smooth muscle cells moving from the intermediate membrane to the intimal membrane, causing fibrous cap thinning and plaque instability. Levels of MMP2, MMP9, TIMP-1 and TIMP-2 are all increased in diabetic patients with dyslipidemia or with acute coronary syndrome [17–19].

Our previous *in vitro* studies suggested reduced β_2_GP I inhibits oxLDL-induced macrophages from forming foam cells and from inducing apoptosis, however β_2_GP I did not have this effect [12]. This raises the question whether early intervention with β_2_GP I and reduced β_2_GP I provides vascular protection in high glucose and high fat animals *in vivo*, and whether β_2_GP I and reduced β_2_GP I can affect MMPs/TIMPs in the aorta. These mechanisms remain unknown and further research is required.

In the current study, diabetic mice models were successfully induced. Following treatment with β_2_GP I and reduced β_2_GP I for three weeks or six weeks, LDL-c in the reduced β_2_GP I group was lower than that in the diabetic control group. Our results suggest that long-term application of reduced β_2_GP I reduces plasma LDL-c levels.

Patients with diabetes are more prone to atherosclerosis and cardiovascular events [3, 4]. In our study, lipid deposition in diabetic mice was demonstrated in the aorta along with atherosclerotic plaques. Total β_2_GP I levels (oxidized and reduced form) in the plasma of stroke patients, and old patients with heart disease, were significantly decreased, and this did not alter after 6 weeks [20]. Further studies have suggested high levels of total β_2_GP I can reduce the risk of myocardial infarction in people older than 60 [21]. In our study, treatment with β_2_GP I resulted in arterial lipid deposition but no plaque formation in blood vessels. However, treatment with reduced β_2_GP I showed that arterial lipid deposition was significantly decreased and plaques in blood vessels had not formed. These results suggest that reduced β_2_GP I can prevent atherosclerosis in diabetic mice.

High glucose levels caused endothelial cells to express higher levels of MMP1, MMP2 and MMP9, however TIMP-1 levels were unaltered *in vitro* [22]. Plasma levels of MMPs and TIMPs change in diabetic patients, but these changes are inconsistent across different studies [23–28]. Papazafiropoulou et al. reported that plasma concentrations of MMP-2 and MMP-9 were not different between diabetic and non-diabetic patients, while TIMP-1 levels were lower in diabetic patients. No significant associations were found between the expression of MMPs and TIMP-1 and arterial stiffness; duration of diabetes emerged as the strongest predictor of arterial stiffness [29]. Uemura reported that diabetes increased the activity of MMP9 via oxidative stress, resulting in increased vascular complications. The probability of these vascular complications occurring was reduced by lowering the activity of MMP9 with antioxidants [30]. In our study, MMP2, MMP9 and TIMP-1 expression in the aortas of diabetic mice were increased. After early intervention with β_2_GP I and reduced β_2_GP I, expression levels of MMP2, MMP9, TIMP-1, and TIMP-2 were decreased, with reduced β_2_GP I having a more pronounced effect. TIMPs are natural inhibitors of MMPs *in vivo* and have anti-atherogenic effects. TIMP-1 overexpression in ApoE-deficient atherosclerotic mice can prevent plaque rupture of vein grafts [31]. The ratio of MMPs to TIMPs have an effect on atherosclerotic processes *in vivo*; TIMP-1 mainly inhibits MMP9, while TIMP-2 mainly inhibits MMP2 [10, 11]. The ratios of MMP2 to TIMP-2 and MMP9 to TIMP-1 are used to represent the total activities of MMPs. In our study, these ratios declined after intervention with reduced β_2_GP I and β_2_GP I.

To explore further vascular protective mechanisms of reduced β_2_GP I, we investigated the p38MAPK signaling pathway. Results from our previous study confirmed that reduced β_2_GP I plays a role in p38MAPK signaling [6]. The promoter upstream of MMPs and TIMPs exists as a cis-acting element and is associated with signaling molecules of the p38MAPK signaling pathway. Activation or inhibition of MMP and TIMP expression is not always consistent [32, 33]. Our findings showed that reduced β_2_GP I down regulated the p38MAPK pathway in aortas.

## Conclusions

Reduced β_2_GP I reduces LDL-c levels, inhibits the formation of plaques, inhibits MMPs/TIMPs ratios in the aorta, and plays a role in vascular protection in diabetic mice. This is probably only one portion of the mechanism(s) involved. MMP2, MMP9, TIMP-1, and TIMP-2 DNA contain GC-rich islands [34, 35], and it is necessary to further investigate whether reduced β_2_GP I can affect methylation of MMPs/TIMPs.

## Abbreviations

β_2_GP I: β_2_-glycoprotein I
MMP: Matrix metalloproteinase
TIMP: Tissue inhibitor of matrix metalloproteinase
STZ: Streptozotocin
p38MAPK: p38 mitogen-activated protein kinase.
